# Versatile control of metal-assisted chemical etching for vertical silicon microwire arrays and their photovoltaic applications

**DOI:** 10.1038/srep11277

**Published:** 2015-06-10

**Authors:** Han-Don Um, Namwoo Kim, Kangmin Lee, Inchan Hwang, Ji Hoon Seo, Young J. Yu, Peter Duane, Munib Wober, Kwanyong Seo

**Affiliations:** 1 Department of Energy Engineering, Ulsan National Institute of Science and Technology (UNIST), Ulsan, 689-798, Korea; 2 Zena Technologies, 174 Haverhill Road, Topsfield, Massachusetts 01983, United States

## Abstract

A systematic study was conducted into the use of metal-assisted chemical etching (MacEtch) to fabricate vertical Si microwire arrays, with several models being studied for the efficient redox reaction of reactants with silicon through a metal catalyst by varying such parameters as the thickness and morphology of the metal film. By optimizing the MacEtch conditions, high-quality vertical Si microwires were successfully fabricated with lengths of up to 23.2 μm, which, when applied in a solar cell, achieved a conversion efficiency of up to 13.0%. These solar cells also exhibited an open-circuit voltage of 547.7 mV, a short-circuit current density of 33.2 mA/cm^2^, and a fill factor of 71.3% by virtue of the enhanced light absorption and effective carrier collection provided by the Si microwires. The use of MacEtch to fabricate high-quality Si microwires therefore presents a unique opportunity to develop cost-effective and highly efficient solar cells.

Vertically aligned silicon microwire (MW) arrays have been extensively investigated as a potential means for developing highly efficient and low cost solar cells^1^,^2^,^3^,^4^,^5^,^6^,^7^,^8^,^9^,^10^,^11^,^12^,^13^,^14^,^15^, as a silicon surface patterned with MW arrays can provide broadband antireflection and enhanced light trapping efficiency^3^,^10^,^11^,^12^,^13^,^15^. Among the many methods developed for the fabrication of Si MW arrays, deep reactive ion etching (DRIE) has proven to provide a satisfactory anisotropic etch profile with a high aspect ratio^4^,^10^,^11^,^12^,^15^,^16^; however, it also produces a rough surface with scallops along the sidewalls of the Si MWs due to its reliance on alternating steps of etching (SF_6_) and passivation (C_4_F_8_)^15^,^17^,^18^,^19^. In addition, plasma-induced surface damage extending from the surface to a depth of up to ~1 μm can reduce the lifetime of minority carriers^20^,^21^,^22^, with Chen *et al.* reporting that RIE-induced surface damage leads to a recombination loss that can degrade short-wavelength response and reduce the open-circuit voltage (*V*_*oc*_). This means that in order to achieve high-efficiency solar cells, the damaged surface must be removed by additional treatment^23^.

As an alternative approach for fabricating Si MW arrays, metal-assisted chemical etching (MacEtch) has attracted great interest because of its simplicity, low fabrication costs, and ability to generate high aspect ratio nanostructures such as nanowires (NWs) and nanoholes^24^,^25^,^26^,^27^,^28^,^29^,^30^,^31^,^32^. Furthermore, as MacEtch is based on a simple redox process in an etching solution, it produces a very smooth and clean Si surface that is free of the surface damage that typically results when using the RIE process. The anisotropic etch profile of the MacEtch process has also made it possible to achieve vertically-aligned Si NW arrays with aspect ratios as high as 220 over large areas^33^. Ordered Si NW arrays have also been successfully fabricated by combining MacEtch and nano-lithography techniques in processes such as interference lithography^34^,^35^ and nanosphere lithography^33^,^36^, but as yet MacEtch has not been directly used for the fabrication of Si MWs with micrometer spacing. Furthermore, although attempts have been made to use MacEtch to fabricate Si microstructures with a high aspect ratio^37^,^38^,^39^,^40^, there are still a number of unresolved issues preventing optimal structures being achieved such as undesired etching, a low etch rate, and surface non-uniformity. Problems can also be encountered as a result of undesired nanostructures being created from voids or fractures in the metal film used as a catalyst^38^,^40^. This means that a MW solar cell with a greater light trapping efficiency than a planar solar cell may still have a lower power conversion efficiency (PCE)^7^ due to the surface non-uniformity created by a non-optimized MacEtch process.

In this paper, we take a systematic look at the mechanism of the MacEtch process in relation to the fabrication of high-quality vertical Si MW arrays. Two mechanism models for efficient redox reaction through a Au catalyst are suggested, with the effect of each model being investigated by controlling the deposition rate and thickness of the catalyst. It is found that the Au film needs to have a thickness of 30 to 40 nm and a fast deposition rate (≥3 Å/s) if high-quality vertically aligned Si MWs are to be obtained without surface damage (i.e., a high etch rate is needed), and so optimization of the Au catalyst structure was used to increase the length of the high-quality Si MWs obtained. These MWs were subsequently used for the fabrication of photovoltaic devices, the performance of which is herein discussed.

## Results

### Possible MacEtch mechanisms for the formation of microscale structures

The MacEtch process consists of two steps. First, nanoparticles or a film of Ag, Au, Pt, Pd, etc. is deposited on a target substrate to provide a catalyst, with films typically being patterned to form an array of holes, wires, or other nano-scale features. Next, the metal-coated substrate is immersed in an etching solution composed of HF and a suitable oxidant such as H_2_O_2_. Chemical reduction of the oxidant on the surface of the metal catalyst generates electrical holes (*h*^*+*^), as represented by Eq. (1), which subsequently leads to dissolution of the Si surrounding the catalyst by HF, as in Eq. (2)^41^,^42^:


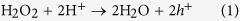



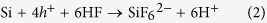


**Figure 1:**
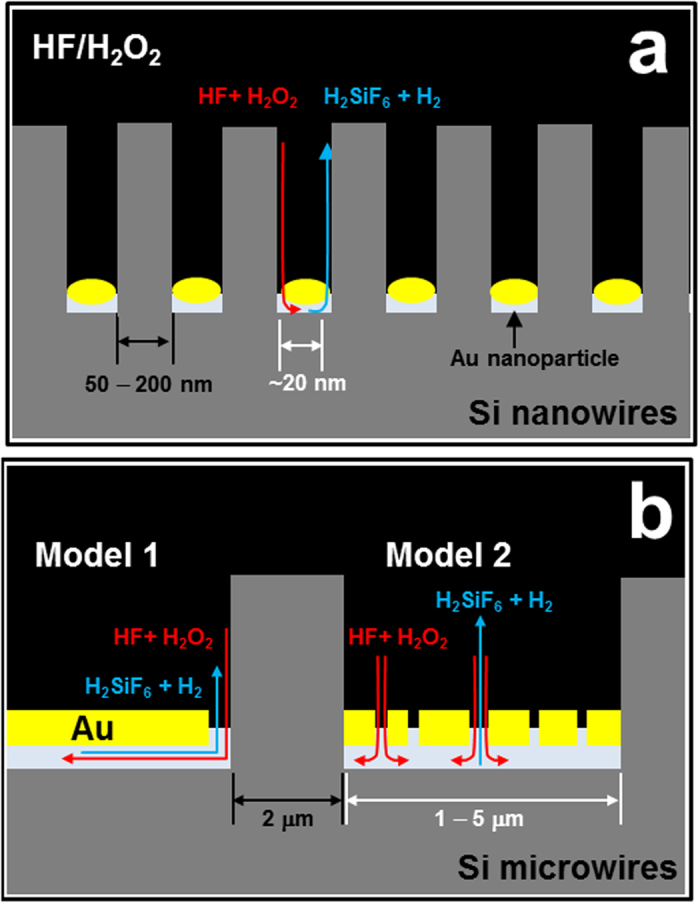
Mechanism models for the redox reactions between Si and reactants in solution though a metal catalyst during MacEtch. (**a**) Formation of Si nanowires: the diffusion of reactants and reaction products occurs through gold nanoparticles. (**b**) Formation of Si microwires: Model 1, in which diffusion takes place in a thin channel formed at the metal/Si interface and Model 2, in which diffusion occurs through small pores in the metal film to the metal/Si interface.

The effectiveness of MacEtch lies in the fact that these redox processes can be selectively localized to only the Si that is directly underneath the metal catalyst. Moreover, as the metal catalyst moves and penetrates into the space formed by the etched Si, the etched structure is ultimately defined by the shape of the catalyst (i.e., the particles or patterned film). Creating Si NWs requires a metal catalyst with a particle-linked structure^43^, and using a nanoparticle diameter of less than ~100 nm ensures that the nanoscale distance (half the nanoparticle diameter) is sufficient to allow fluent diffusion of the etchant and dissolved Si (Eq. (2)) along the Au/Si interface. Under such conditions, which are illustrated in Fig. 1a, the redox reaction of MacEtch occurs immediately at the Au/Si interface in contact with the etchant. If, on the other hand, a micrometer-scale Si structure is to be created by MacEtch, then a continuous Au film is needed rather than Au nanoparticles. In the case of the continuous Au film shown in Fig. 1b, the catalytic reaction of MacEtch is expected to follow a very different mechanism to that of a particle-linked catalyst. For instance, the etching solution is no longer able to diffuse beneath or penetrate into the continuous film, and for this reason the mechanism of the redox reactions needs to be reconsidered and systematically investigated for the etching of vertical Si MWs.

We suggest two possible models for the diffusion of reactants and reaction products during the MacEtch of Si MWs, both of which are illustrated in Fig. 1b. In Model 1, reactants (HF and H_2_O_2_) initially diffuse into the metal/Si interface, with the subsequent diffusion of reaction products (e.g., H_2_SiF_6_ and H_2_ gas) taking place in a thin channel formed at the metal/Si interface. In contrast, diffusion in Model 2 occurs via small pores in the metal film leading to the metal/Si interface, which is where the Si atoms are oxidized and etched away. A series of methodical experiments were performed as part of this study to elucidate which of these models is the more dominant in micrometer scale MacEtching, and based on the results, we propose a new combined mechanism and optimized process parameters for the formation of vertical Si MWs with high aspect ratio by MacEtch.

### Effect of metal catalyst thickness and spacing

**Figure 2:**
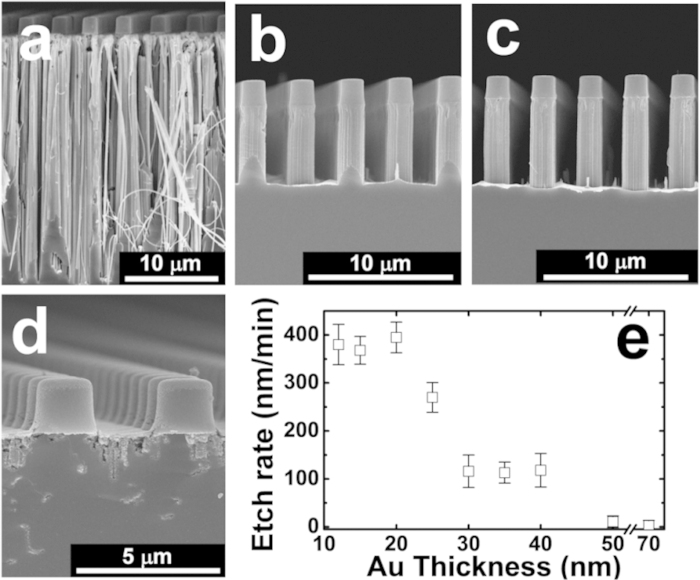
SEM images of microwires etched from a substrate with a (**a**) 15, (**b**) 30, (**c**) 40, and (**d**) 50 nm-thick Au film. (**e**) Variation in etching rate as a function of Au film thickness. The well-aligned vertical microwires were obtained using an Au film with a thickness of 30 to 50 nm.

Differences between Model 1 and 2 in terms of their change in etching rate as a function of the metal catalyst thickness and spacing (i.e., the distance between MWs) can be attributed to the diffusion distance (*D*_*diff*_) of the reactants (HF and H_2_O_2_) and reaction products (H_2_SiF_6_ and H_2_ gas). That is, in Model 1 *D*_*diff*_ is largely dependent on the spacing, whereas in Model 2 it is governed by the porosity of the metal film. To test the notion that the etch rate should not be effected by film thickness if Model 1 is dominant, metal films with different thicknesses were deposited on Si substrates using the same photoresist pattern (2 μm in diameter and 2 μm spacing). The SEM images in Fig. 2 show the wires that were etched using Au film thicknesses (*t*_*Au*_) of 15 to 70 nm; a *t*_*Au*_ of less than 10 nm found to result in random and disordered etching of the Si (see supplementary Fig. S1a). All samples were immersed in an etching solution containing 10 M HF and 0.3 M H_2_O_2_ for 60 min, but in order to more clearly determine the etch rate, the photoresist dots on the tips of each MW were not removed prior to MacEtching. As shown in Fig. 2a, the etched structures appear to evolve from pores or wall-like structures into wires with a diameter in the range of 50–200 nm with increasing *t*_*Au*_ (15 nm < *t*_*Au*_ < 20 nm), which can be explained by the fact that very thin Au films (*t*_*Au*_ < 30 nm) consist of isolated nanoparticle structures with wide gaps and weak interconnections between them (see supplementary Fig. S1b). The use of a thick Au film (30 nm ≤ *t*_*Au*_ < 50 nm), on the other hand, results in the ordered MWs seen in Fig. 2b,c. Thus, with increasing *t*_*Au*_, the deposited nanoparticles tend to form interconnected networks that shrink the interspace between them, causing them to cover almost the entire surface of the substrate. However, when the thickness reaches 50 nm or more (Fig. 2d), neither Si NWs nor MWs are formed. The variation in etch rate with Au film thickness shown in Fig. 2e interestingly reveals that the etch rate of a 40 nm-thick Au film (118 nm/min) is much the same as that of a 30 nm-thick Au film (113 nm/min), despite the fact that the increase in thickness coincides with a decrease in pore density. This therefore provides at least indirect evidence that Model 1 is the dominant mechanism.

The reliability of Model 1 was investigated by depositing metal films of the same thickness, but with different spacing, onto Si substrates. That is, since *D*_*diff*_ depends only on the spacing according to Model 1, the etch rate should decrease with increased spacing. Identical MacEtch conditions were used with Si MW spacings of 2 μm and 5 μm, with the SEM images shown in supplementary Fig. S2 confirming that the etch rate did indeed vary in relation to the spacing. Specifically, the etch rate with a 2 μm spacing (136 ± 15 nm/min) was notably higher than that with a 5 μm spacing (94 ± 30 nm/min). In addition, the etch rate at the centre of the 5 μm gap was comparatively slower than that at the edge of the Au film (the Au/photoresist interface), which caused the Au film to bend (see Fig. S2c). This non-uniformity in etch rate can be attributed mainly to the long *D*_*diff*_ that the etchant needs to travel in order to pass beneath the Au film, and means that the etch rate decreases with increasing spacing of the Au film; a conclusion that supports the notion that chemical diffusion is ruled by Model 1.

### Reaction kinetics of MacEtch for Si MWs

**Figure 3:**
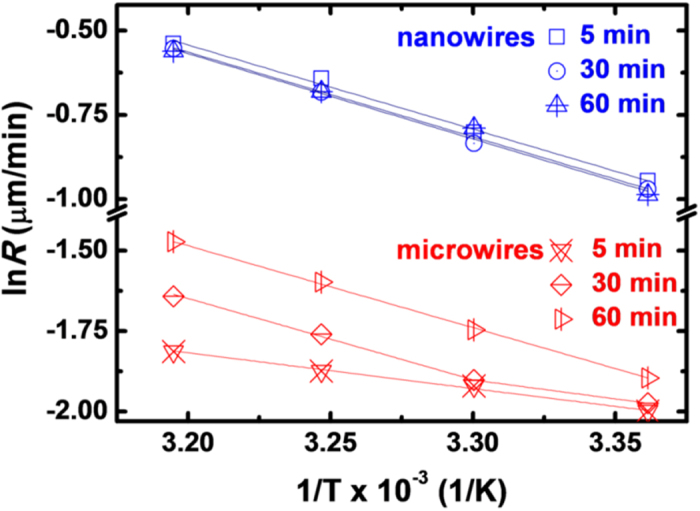
Arrhenius plots showing the temperature dependence of the etch rate (ln(rate) *vs* 1/T) of Si nanowires (blue symbols) and microwires (red symbols) during 5, 30, and 60 min of MacEtching. The solid lines represent best fits to the experimental data. The average activation energies for the Si nanowires and microwires were calculated from the Arrhenius plots.

If the etching rate of Si MWs is indeed determined by the distance of chemical diffusion along the thin channel between the metal catalyst and Si surface, then the etching kinetics are clearly different from those of Si NWs in which the redox reaction occurs as soon as the Au nanoparticle/Si interface makes contact with the etchant. To better understand the etching reaction kinetics of Si MWs, the temperature dependence of the etch rate was investigated by means of Arrhenius plots of ln(*R*) = ln(*A*) − *E*_*a*_/(*k*_*B*_*T*), where *R* is the etch rate, *A* the pre-exponential (frequency) factor, *E*_*a*_ the activation energy required for etching, and *k*_*B*_ the Boltzmann constant. Based on this analysis, the average activation energy for the Si MWs was found to be much smaller than that of Si NWs, with *E*_*a*_^*MW*^ = 0.15 ± 0.05 eV and *E*_*a*_^*NW*^ = 0.36 ± 0.01 eV when an etching time of 5 min was used (Fig. 3). The activation energy for the Si NWs is similar to previously reported values^44^,^45^, and so clearly the overall rate of MacEtching is determined by the interplay between the two competing events: chemical diffusion along the Au/Si interface, and the Au-catalyzed redox reaction between the Si and etching solution. Given that the etching rate of the Si NWs is determined by a redox reaction with a relatively short *D*_*diff*_ (i.e., less than the diameter of the Au nanoparticles), the activation energy for etching corresponds to the energy barrier for displacing a Si atom^44^. In contrast, the long chemical diffusion through a very thin channel between the Au film and Si in the MacEtching of Si MWs only provides a very limited amount of the reactant required for redox. In other words, the etching rate of Si MWs is determined by the migration of reactants through the electrolyte and their interaction^46^,^47^,^48^, not the kinetics of the redox reaction. Consequently, the activation energy should approximate that required for chemical diffusion^49^, and this is lower than that required for surface-controlled reactions^50^.

The etch rate and activation energy was further investigated by increasing the etching time up to 30 and 60 min, as shown in Fig. 3. It is evident from this that while the activation energy for Si MWs increases from 0.16 ± 0.07 to 0.34 ± 0.04 eV at an elevated temperature (≥30 °C), it remains constant at 0.36 ± 0.03 eV in the case of Si NWs. During the rapid etching of Si MWs at elevated temperature, the continuous metal film should readily form a discontinuous film and individual nanoparticles due to the non-uniform etching rate over its surface (see supplementary Fig. S3). These isolated particles should, in turn, increase the rate at which the Si NWs between the Si MWs are etched, as the short *D*_*diff*_ means that the etching rate is determined by the redox reaction of the Si NWs. The average activation energy for the randomly-etched MWs at elevated temperature (0.35 ± 0.03 eV) turned out to be similar to that for vertical Si NWs (0.36 ± 0.01 eV), indicating that the etching mechanism of the randomly-etched MWs is indeed similar to that of Si NWs due to the short *D*_*diff*_.

**Figure 4:**
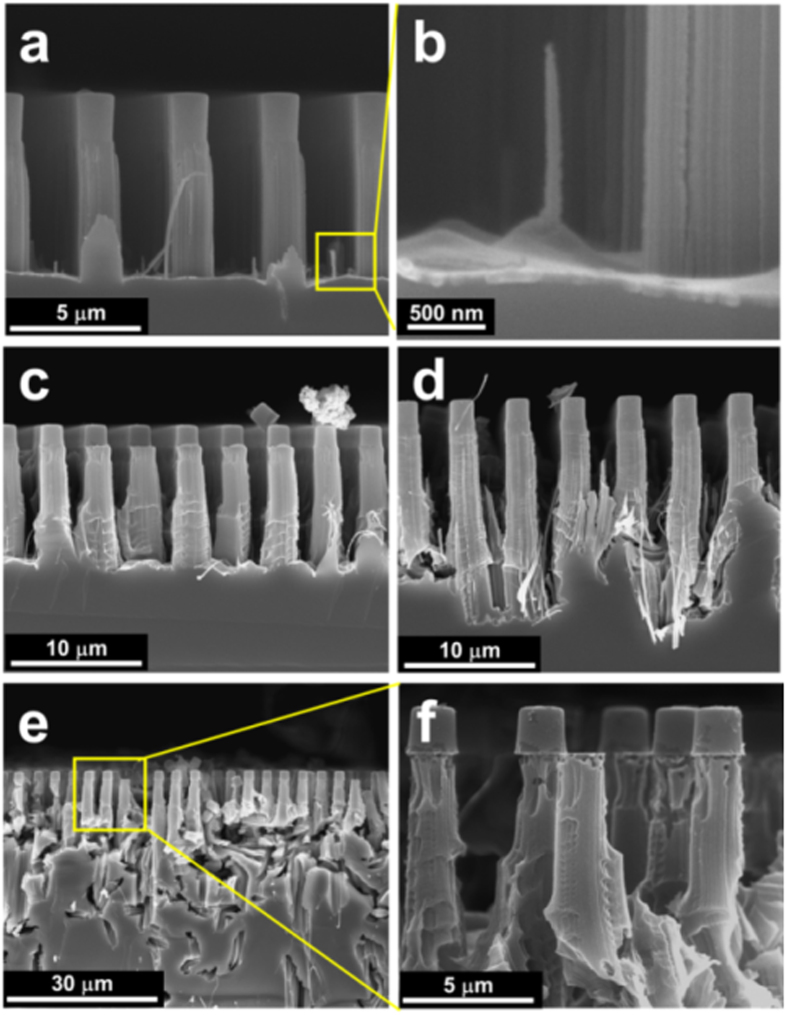
SEM images of Si microwires etched at 30 °C for (**a**,**b**) 30, (**c**) 45, (**d**) 60, and (**e**,**f**) 90 min. In panel (**b**), the magnified image of the region marked in panel (**a**) shows the undesired formation of nanowires between Si microwires. With etching time greater than 50 min, a discontinuous and cracked film was produced that resulted in the formation of randomly etched structures.

In the case of Si MWs with a high aspect ratio, the etching time should increase without changing the morphology of the metal film. Figure 4 shows SEM images obtained of Si MWs etched for various times ranging from 30 to 90 min, from which we see that during the initial stage of the MacEtch process (≤20 min), the continuous Au film produces vertical Si MW arrays at an etch rate of ~130 nm/min. With increasing time, however, the continuous Au film is likely to crack due to non-uniform etch rate between the edge and centre of the film, even if MacEtching is conducted at room temperature. These discontinuous and cracked films tend to have small pores, which lead to the undesired formation of NWs between the Si MWs (Fig. 4a,b). With etching times longer than 60 min, a discontinuous Au film is produced that results in randomly etched structures at the bottom of the Si MWs, as can be seen in Fig. 4e,f. These random structures can be explained by the fact that excess holes generated in the isolated Au particles can etch Si atoms that exist in crystal planes in which there is a greater extent of inter-atomic bonding^44^, which means that MacEtching occurs preferentially along the <110> or <111> direction. Thus, in order to prevent the formation of the isolated Au particles, the difference in etching rate between the centre and edge of the film should be minimized.

### A combined model for Si MWs with a high aspect ratio

**Figure 5:**
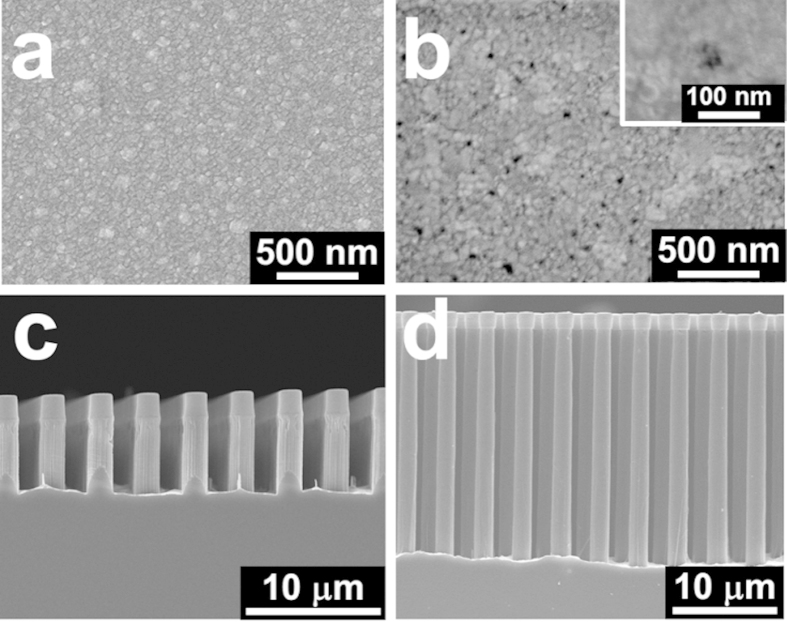
Top-view SEM images of 30 nm-thick Au films deposited at rates of (**a**) 1 and (**b**) 3 Å/s. The inset of (**b**) shows a high-magnification SEM image of a pore in the Au film. Cross-sectional SEM images of Si microwires etched from a substrate with a 30 nm-thick Au film deposited at a rate of (**c**) 1 and (**d**) 3 Å/s. A fast Au film deposition rate produced small pores, leading to a higher Si microwire etch rate of ~386 nm/min due to the shorter chemical diffusion path.

There are a number of key issues such as the slow, non-uniform etch rate and the deformation of the Au film that prevent the reproducible fabrication of Si MWs with a high aspect ratio due to the long chemical diffusion in Model 1. However, if diffusion occurs instead via the small pores in the metal film proposed in Model 2, then these issues can be resolved simply by reducing the *D*_*diff*_. To increase the etch rate to form MWs with a high aspect ratio of more than 10, the morphology of the Au film was modified by introducing pores. This was based on the premise that the morphology of a thin film is typically determined by its deposition rate, in that nuclei are formed at certain cites then grow via surface diffusion and direct impingement to create larger and more irregular islands. In other words, continuous deposition creates a metallic network with more regular and smaller voids, and eventually a uniform metal layer is created^51^,^52^. At low deposition rates, the density of nuclei capable of merging to form grains is small, leading to a dense film with a small grain size. In contrast, a higher deposition rate increases the number of metal atoms deposited over a given period of time, resulting in the formation of bigger grains^53^. Thus, deposition rates of 0.1 to 3 Å/s and Au thicknesses of 20 to 40 nm were used to modify the morphology of the Au film (see supplementary Fig. S4); the SEM images in Fig. 5 showing the morphologies achieved and corresponding etching results. It is apparent from this that while pores are created by high-rate deposition (Fig. 5b), no pores can be seen in the Au film deposited at a lower rate (Fig. 5a). Moreover, there is a significant increase in the etching rate of the film over a period of 60 min if it is created using a higher rate of deposition, as shown in Fig. 5c,d, with the 23.2 μm final length of the etched Si MWs corresponding to an etch rate of ~386 nm/min. This etch rate is only slightly less than the ~397 min/min of Si NWs, which suggests that the pores formed during the high-rate deposition of the Au film can act as channels to reduce *D*_*diff*_ and increase the etch rate without any discernible deformation of the film.

**Figure 6:**
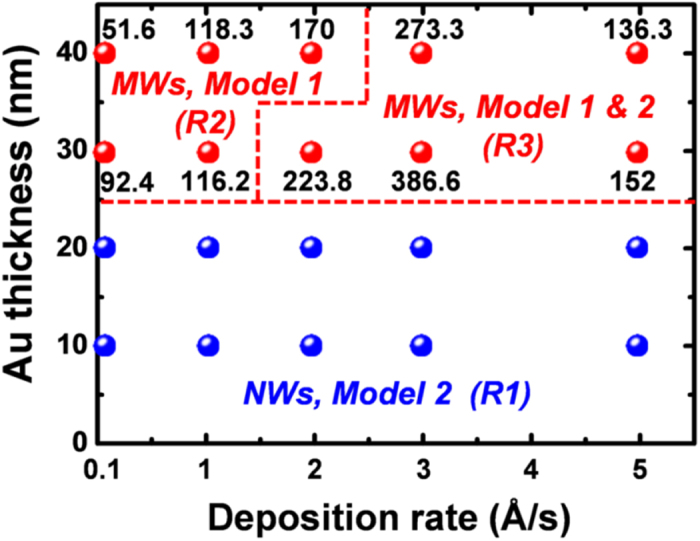
Wire structures and MacEtch rates as a function of the deposition rate and thickness of the Au catalyst film. MacEtch conditions can be divided into three distinct regions. In Region 1 (R1, blue circles at bottom), Si nanowires can be obtained due to the fact that the Au film consists of a network of isolated nanoparticles. The conditions of Region 2 (R2, red circles at top left) are sufficient to produce Si microwires, but high aspect ratio structures cannot be obtained due to the non-uniform etch rate. In Region 3 (R3, red circles a top right), the vertically aligned Si microwires with a high aspect ratio were obtained using an Au film with a thickness of 30 to 40 nm and a fast deposition rate (≥3 Å/s) in Region 3.

Figure 6 summarizes the conditions used for the etching of Si in relation to the Au thickness and deposition rate achieved, which can be broadly divided three distinct regions. In Region 1 (R1), Si NWs can be obtained due to the fact that the Au film consists of a network of isolated nanoparticles that do not completely cover the surface of the Si, and so a high etch rate is possible due to the short *D*_*diff*_ of Model 2. The creation of Si MWs, however, requires that the Au film thickness be at least 30 nm in order to ensure complete coverage of the Si surface. The conditions of Region 2 (R2) are sufficient to produce Si MWs, but high aspect ratio structures cannot be obtained due to the formation of isolated Au particles as a result of the non-uniform etch rate. Further complicating matters is the fact that the etch rate is very low due to the long *D*_*diff*_ of Model 1, unless of course the morphology of the Au film is suitably modified. Between Region 1 and 2, however, there is a clear processing window (Region 3, R3) in which high quality Si MWs with a high aspect ratio can be obtained via the MacEtch process. This region is marked in Fig. 6, which shows that the appropriate ranges for the Au thickness and deposition rate are 30–40 nm and 2–5 Å/s. Furthermore, this represents a region in which long-range diffusion by Model 1 and short-range diffusion by Model 2 coexist. This means that in order to achieve a rapid rate of etching, it is simply a matter of ensuring the dominance of Model 2 by creating small pores in a continuous film deposited at a high rate (≥3 Å/s). A complete summary of these results and corresponding SEM images can be found in supplementary Fig. S5.

### MacEtched Si microwire solar cells

**Figure 7:**
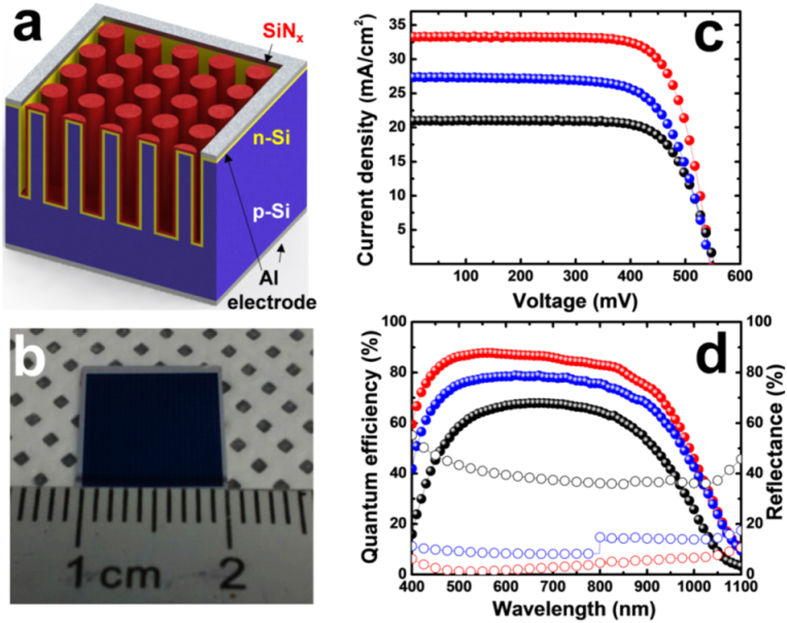
(**a**) Schematic illustration and (**b**) optical image of Si microwire solar cells. (**c**) Current density versus voltage characteristics of planar (black circle and line) and microwire solar cells with (red circle and line) and without a thin SiN_x_ layer (blue circle and line) under an illumination of AM 1.5G. (**d**) External quantum efficiencies (solid circle) and reflectance spectra (open circle) of planar (black circle and line) and microwire cells with (red circle and line) and without thin SiN_x_ layer (blue circle and line). The microwire solar cells with a SiN_x_ layer achieved a photovoltaic conversion efficiency of 13.0% owing to a significant improvement in external quantum efficiency and short-circuit current density.

High quality vertical Si MWs with a diameter and spacing of 2 μm, and a 10 μm length, were fabricated on a 4-in. wafer using optimized MacEtch conditions to assess their suitability for use in solar cell applications (Fig. 7). For this, a highly doped n-type emitter layer was first added using a spin-on-doping (SOD) method^10^ to provide a radial p-n junction with a sheet resistance of ~30 Ω/sq in which the junction depth was estimated to be ~540 nm from the surface and the surface doping concentration measured to be 7.1 × 10^20^ cm^−3^ based on its secondary ion mass spectrometry (SIMS) profile (supplementary Fig. S6). A thin SiN_x_ layer (60-nm-thick) was then deposited by plasma-enhanced chemical deposition (PECVD) to provide an anti-reflection and passivation layer. Table 1 shows the photovoltaic properties of planar and MW solar cells. This reveals that the best performance of the MW solar cells achieved a photovoltaic conversion efficiency (*PCE*) of 13.0%, along with an open-circuit voltage (*V*_*oc*_), short-circuit current density (*J*_*sc*_), and fill factor (*FF*) of 547.7 mV, 33.2 mA/cm^2^, and 71.3%, respectively. Note that the *PCE* of the Si MW solar cell is still relatively low compared to that of conventional crystalline Si solar cells, a possible reason for which would be non-optimized doping of emitter layer and top electrode structure. That is, since the front contact was formed by selectively-patterned Al electrode around the Si MW arrays with 1 cm^2^ cell area as shown in Fig. 7, the MW solar cells should have a highly-doped emitter for efficient collection of carriers through the front electrode, leading to serious Auger and surface recombination in the MWs. Despite this performance degradation due to recombination, the *J*_*sc*_ value of the MW solar cells represents a significant improvement over that of the planar devices (21.0 mA/cm^2^), which can mainly be attributed to the enhanced light trapping efficiency of the vertically-aligned MWs. The reflectance (Fig. 7d), wavelength-averaged over the main spectral range from 400 to 1000 nm, is also reduced from ~39.9 to ~3.5% when highly-dense MWs are used, which is consistent with the increase in external quantum efficiency (EQE in Fig. 7d). Furthermore, the improved EQE value at short-wavelengths (400–550 nm) indicates a much better response to blue light (high energy photons) than a planar device that is caused not only by the increased light absorption, but also the effective carrier separation of the radial junction in MWs. The *V*_*oc*_ and *FF* values of the MW solar cells are comparable to those of the planar device, even though the MW cells do have a much larger surface area, which can be explained through an analysis of the effective minority carrier lifetime (τ_eff_). As shown in supplementary Fig. S7, the effective lifetimes of the two cell types were measured by a microwave photoconductivity decay (μ-PCD) method using a commercially available scanner (Semilab, WT-2000PVN), which found that the τ_eff_ of the MW cells (9.63 μs) is very similar to that of the planar cells (9.98 μs). On the basis of this, it is considered that the high-quality Si MWs fabricated by optimized MacEtch conditions can produce highly efficient solar cells.

## Discussion

Experimental investigation of the mechanism behind which Si MWs can be created by the MacEtch process has demonstrated through a series of experiments that this process can be controlled through varying the thickness (10 to 70 nm) and deposition rate (0.1 to 5 Å/s) of the thin Au catalyst layer. In other words, a thick (30 to 50 nm) Au film produces well-aligned vertical Si MW arrays. Based on Arrhenius plots, the activation energy for the Si MWs (0.15 ± 0.05 eV) was found to be much smaller than that of Si NWs (0.36 ± 0.01 eV), indicating that the etching kinetics of Si MWs should be different from those of Si NWs. It was also found that the diffusion distance of the reactants and reaction products needs to be of a nanometer-scale to ensure a high etch rate, and by reducing the diffusion distance, high-quality Si MWs with a high-aspect ratio of up to 11.6 (2 μm in diameter and 23.2 μm in length) were successfully obtained. The rapid rate of etching could be demonstrated by creating small pores in the continuous film deposited at the high rate of ≥3 Å/s. Using this knowledge, highly efficient (13.0%) MW solar cells were fabricated via the MacEtch method, with a *V*_*oc*_ of 547.7 mV, a *J*_*sc*_ of 33.2 mA/cm^2^, and a FF of 71.3% all being achieved as a result of the enhanced light trapping of the vertically aligned wire arrays and the effective carrier collection of the high-quality radial junction. These MacEtched MWs therefore clearly represent a very promising candidate for the next-generation of cost-effective photovoltaics.

## Methods

### Fabrication and characterization of vertical Si microwires

Si MWs arrays were fabricated from Czochralski (CZ) p-type Si wafers (resistivity of 1–10 Ω.cm, 550-μm thick). Circular-shaped photoresist dot arrays (2 μm in diameter, 2 or 5 μm spacing) were periodically patterned using DNR-L300-30 photoresist (Dongjin Semichem) through the photolithography. To remove photoresist residues, the oxygen-plasma treatment were conducted in plasma asher (V15-G, KAMI) with 300 W for 5 min. Then, thin Au films were uniformly deposited on the Si substrates by thermal evaporator. The thickness and deposition rate were varied to control the morphology of Au film. MacEtch in a mixed solution of de-ionized water, HF (10 M), and H_2_O_2_ (0.3 M) resulted in the formation of NWs or MWs depending on metal catalyst conditions. After MacEtch process, Au films were removed using the commercial Au etchant (Sigma-aldrich) and then the substrates were cleaned in acetone to remove all photoresist remaining on top of the MWs. The surface morphology of Si MWs was characterized by field-emission scanning electron microscopy (FE-SEM, Hitachi S-4800).

### Fabrication of vertical Si microwire solar cells

An emitter layer was formed by phosphorus diffusion via the spin-on-dopant (SOD) method. First, phosphorus dopant source (P509, Filmtronics, Inc.) was spin-coated on a dummy Si wafer, and then baked at 200 °C for 10 min. To form the conformal doping on MWs, we positioned Si MWs sample so that it faced the phosphorus-coated dummy wafer. The diffusion doping was carried out in a tube furnace under a mixed ambient of 20% O_2_ and 80% N_2_ at 850 °C. Phosphorus glass that remained after the SOD diffusion was removed by using a diluted HF solution. After removing phosphorus glass and SiO_x_ layer, a thin SiN_x_ layer (60-nm-thick) was deposited by PE-CVD (PEH-600, SORONA). For the top and bottom contacts, 200-nm-thick Al films were deposited on the top and bottom of samples using a thermal evaporator. In creating the top electrode with the optical widows, the Si MWs were covered with photoresist (AZ9260, AZ electronic materials, thickness of ~20 μm) before the metal deposition using lithography process. The active area of the MW solar cells was 1 cm^2^.

### Characterization of vertical Si microwires solar cell

The depth profile of phosphorus ions has been measured by using a magnetic-sector secondary ion mass spectrometer (SIMS, CAMECA IMS 7 f) attached a Cs ionization source. The Cs^+^primary ions were accelerated to 10 keV and the secondary positive ions were extracted at 5 keV. Current-voltage (*I-V*) characteristics of the devices in the dark were investigated using a semiconductor parameter analyzer (4200-CSC, Keithley). The photovoltaic properties of our solar cells were investigated using a solar simulator (Class AAA, Oriel Sol3A, Newport) under AM 1.5G illumination. Incident flux was measured using a calibrated power meter, and double-checked using a NREL-calibrated solar cell (PV Measurements, Inc.). EQE was measured using a Xe light source and a monochromator in the wavelengths range of 400–1100 nm. Optical reflection measurements were performed over wavelengths of 400–1100 nm using a UV-Vis/NIR spectrophotometer (Cary 5000, Agilent) equipped with a 110 mm integrating sphere to account for total light (diffuse and specular) reflected from the samples. The effective lifetimes were measured by the microwave photoconductivity decay (μ-PCD) method using a commercially available lifetime scanner (Semilab, WT-2000PVN). In this method, the samples are illuminated by a laser pulse (905 ± 10 nm pulsed laser operating at 200 nm cycles) and then the decay of minority carriers is measured by monitoring the reflected microwave signal.

## Additional Information

**How to cite this article**: Um, H.-D. *et al.* Versatile control of metal-assisted chemical etching for vertical silicon microwire arrays and their photovoltaic applications. *Sci. Rep.* **5**, 11277; doi: 10.1038/srep11277 (2015).

## Supplementary Material

Supplementary Information

## Figures and Tables

**Table 1:** Photovoltaic performance of planar and MW solar cells with and without thin SiN_x_ layer.

	***V*_*oc*_ [mV]**	***J*_*sc*_ [mA/cm^2^]**	***FF* [%]**	***PCE* [%]**
Planar cell	557.8.	21.0	72.6	8.5
Si MW cell w/o SiN_x_	547.5	27.3	70.7	10.6
Si MW cell w/ SiN_x_	547.7	33.2	71.3	13.0
