# Supplementary material for: Versatile control of metal-assisted chemical etching for vertical silicon microwire arrays and their photovoltaic applications
Source: Sci Rep. 2015 Jun 10;5:11277. doi: 10.1038/srep11277 (PMC4462046; doi:10.1038/srep11277)
Supplement: Supplementary Information [file srep11277-s1.doc]

**SUPPLEMENTARY INFORMATION**

**Versatile Control of Metal-Assisted Chemical Etching for Vertical Silicon Microwire Arrays and Their Photovoltaic Applications**

Han-Don Um^1^, Namwoo Kim^1^, Kangmin Lee^1^, Inchan Hwang^1^, Ji Hoon Seo^1^, Young J. Yu^2^, Peter Duane^2^, Munib Wober^2^ and Kwanyong Seo^1^*

^1^Department of Energy Engineering, Ulsan National Institute of Science and Technology (UNIST), Ulsan, 689-798, Korea

^2^Zena Technologies, 174 Haverhill Road, Topsfield, Massachusetts 01983, United States

* Corresponding authors: kseo@unist.ac.kr


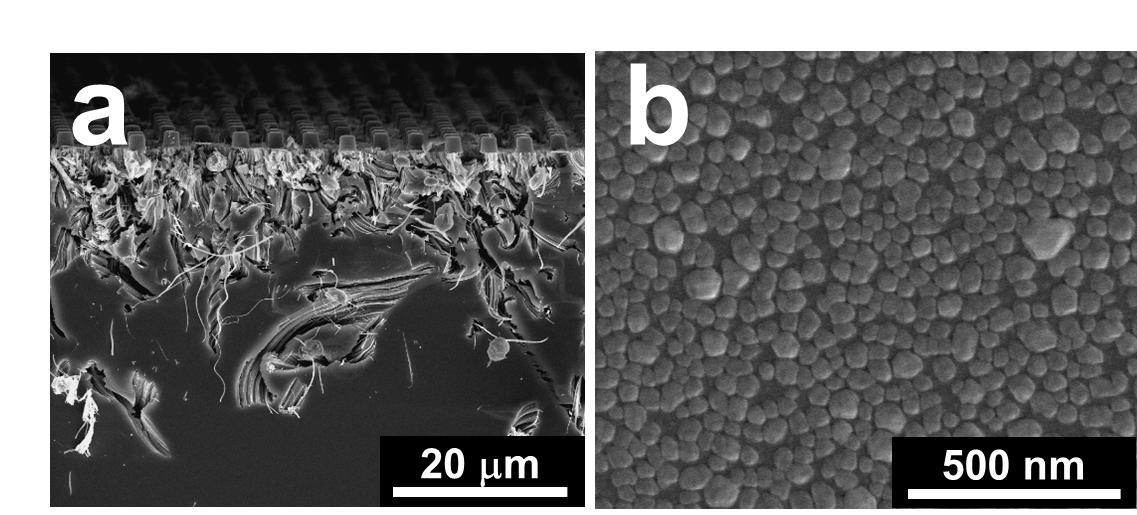


**Fig. S1** (a) Cross-sectional SEM image of Si microwires over-etched from a substrate with a 10-nm-thick Au film consisting of nanoparticles. (b) Plan-view SEM image of the Au film.


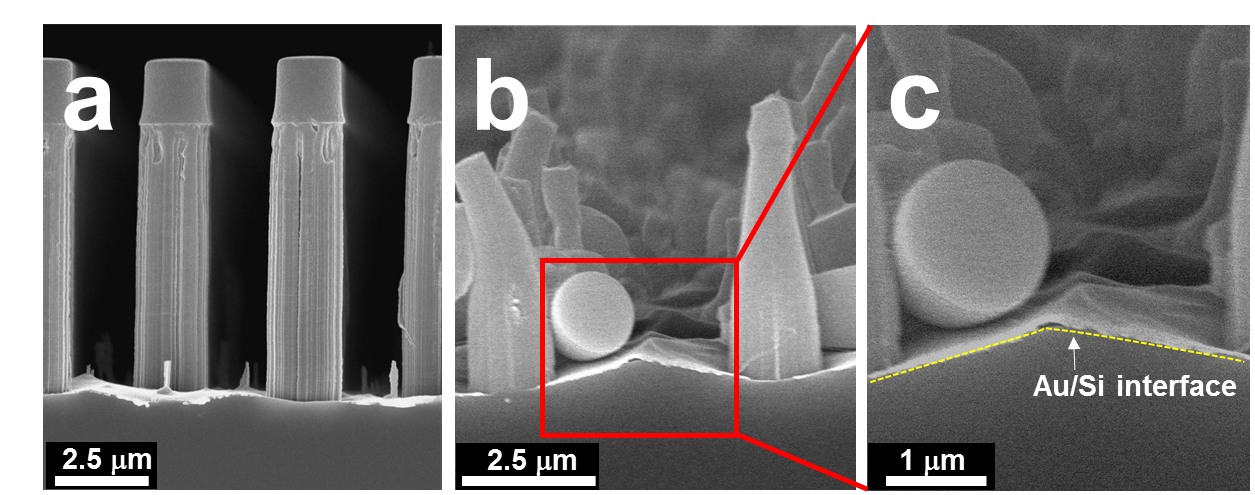


**Fig. S2** SEM images of microwires with (a) 2 μm and (b) 5 μm spacing. (c) Magnified image of the region marked in panel (b). The non-uniformity in etch rate can be mainly attributed to the long diffusion path that the etchant needs to travel in order to pass beneath the Au film, which means that the etch rate decreases with increasing Au film spacing.

**Fig. S3** (a) SEM image of microwires with a discontinuous Au film. (b) Magnified image of the region marked in (a), which clearly shows the discontinuous Au film between the Si microwires. (c) SEM image of randomly-etched microwires with isolated Au films. The bright regions marked by arrows represent isolated Au films formed during MacEtching. The discontinuous and isolated Au films produced randomly etched structures at the bottom of the Si microwires.

**Fig. S4** Top-view SEM images of 40-nm-thick Au films deposited at rates of (a) 0.1 and (b) 3 Å/s, and 20-nm-thick Au films deposited at rates of (c) 0.1 and (d) 3 Å/s. The small pores in the continuous film were formed by a high deposition rate of ≥ 3 Å/s.


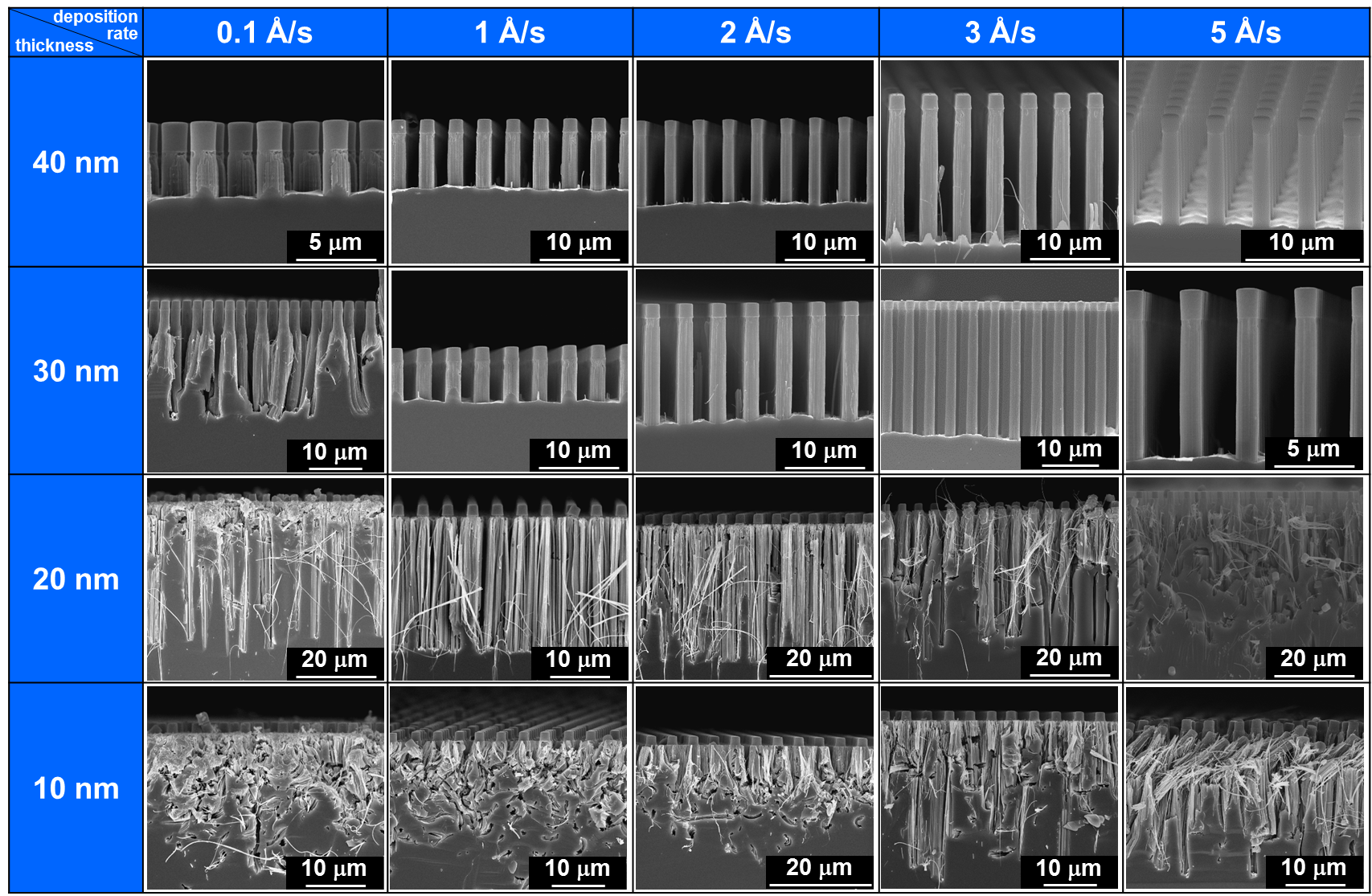


**Fig. S5** Cross-sectional SEM images of Si structures with various thicknesses and Au film deposition rates. This demonstrates a clear processing window in which high quality Si microwires with a high aspect ratio can be obtained via the MacEtch process. The appropriate ranges for the Au thickness and deposition rate are 30−40 nm and 2−5 Å/s, respectively.

**Fig. S6** Secondary ion mass spectrometry depth profile for phosphorus annealed at 850 °C. The junction depth was estimated to be ~540 nm, and the surface concentration was measured as 7.1 × 10^20^ cm^−3^.


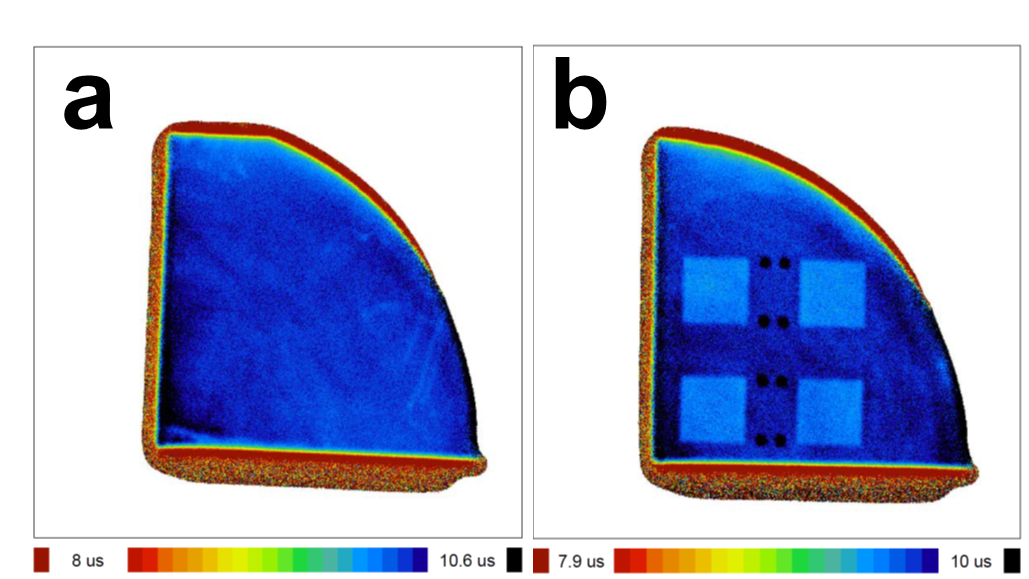


**Fig. S7** Minority-carrier lifetime maps of (a) planar and (b) microwire cells. The effective minority carrier lifetime of the microwires cells (9.63 μs) is very similar to that of the planar cells (9.98 μs), indicating that the high-quality Si microwires fabricated under optimized MacEtch conditions can produce highly efficient solar cells.
